# The Need of Drainage After Cholecystectomy

**DOI:** 10.1155/1990/37926

**Published:** 1990

**Authors:** Julio A. Diez, M. Raúl Pujato, Alberto R. Ferreres

**Affiliations:** 1 Gastroenterology section Department of Surgery Hospital de Clinicas “José de San Martín” University of Buenos Aires Buenos Aires Argentina; 2 Gastroenterology section Department of Surgery Hospital de Clinicas “José de San Martín” University of Buenos Aires, Av. Córdoba 2351 Buenos Aires 1120 Argentina

## Abstract

In an attempt to rationalize the use of intraperitoneal drainage of the subhepatic space after simple, elective cholecystectomy, a prospective study was designed to compare the post-operative course with and without drainage. There was a higher incidence of postoperative fever of unknown origin and wound infection in the drained group. In the group without drainage the hospital postoperative stay was shorter and there were no complications. The results suggest that routine surgical drainage after uncomplicated cholecystectomy is unnecessary and could be a source of postoperative fever and a higher incidence of wound infection.


*HPB Surgery*, 1990, Vol. 3, pp. 5–10
Reprints available directly from the publisher
Photocopying permitted by license only

© 1990 Harwood Academic Publishers GmbH
Printed in the United Kingdom

### THE NEED OF DRAINAGE AFTER CHOLECYSTECTOMY

JULIO A. DIEZ*, M. RAÚL PUJATO and ALBERTO R. FERRERES
*Gastroenterology section, Department of Surgery, Hospital de Clinicas "José de San Martín", University of Buenos Aires, Buenos Aires.*

*(Received 9 October 1989)*

In an attempt to rationalize the use of intraperitoneal drainage of the subhepatic space after simple, elective cholecystectomy, a prospective study was designed to compare the post-operative course with and without drainage. There was a higher incidence of postoperative fever of unknown origin and wound infection in the drained group. In the group without drainage the hospital postoperative stay was shorter and there were no complications. The results suggest that routine surgical drainage after uncomplicated cholecystectomy is unnecessary and could be a source of postoperative fever and a higher incidence of wound infection.

KEY WORDS: Cholecystectomy, drainage, wound infection

### INTRODUCTION

In 1919, thirty one years after Langenbuch[1] performed the first cholecystectomy, cholecystectomy without drainage was introduced in Germany and referred to as "the ideal cholecystectomy"[2]. Since then, sporadic but favorable reports have preferred the omission of drains[3,4,5,6]. Easier convalescence, decreased rate of complications and shortened hospital stay were the advantages mentioned. The effectiveness of drains in forestalling the collection of bile and blood is in dispute. When such complications have been reported, they have invariably occurred in instances where drains were employed[7]. Nonetheless 90% of surgeons routinely use drainage after simple, elective cholecystectomy.

In an attempt to rationalize the use of drains, a prospective study was devised to investigate whether routine drainage after simple and uncomplicated cholecystectomy is imperative, through the comparison of the postoperative course.

### METHODS

From May 1985 to April 1987 a total of 200 patients underwent elective cholecystectomy at the Department of Surgery of the Hospital de Clínicas "José de San Martín" of the University of Buenos Aires.

*Correspondence to: J.A. Diez, Gastroenterology Section, Department of Surgery, Hospital de Clínicas "José de San Martín", University of Buenos Aires, Av. Córdoba 2351, (1120) Buenos Aires, ARGENTINA

The age of the patients ranged from 16 to 74 (mean 56.28) and 153 (76.5%) were women. All the operations were performed by residents under the supervision of staff surgeons and informed consent was provided in every case. The surgical technique was: right subcostal incision, operative cholangiogram through cannulation of the cystic duct, cholecystectomy, suture of the gallbladder bed with atraumatic 2/0 chromic catgut, closure of the abdominal wall in two layers with continuous suture of polyglycocolic acid (Dexon$^R$).

In the opinion of the surgical teams the operation was regarded as simple in 148 patients (74%), difficult in 47 (23.5%) and very difficult in 5 (2.5%). Excluded from the study were patients in whom common bile duct stones were detected and who underwent common bile duct exploration and T-tube drainage. None of the patients received antibiotic prophylaxis, as this is the policy in the Department for elective cholecystectomy without preceding jaundice or acute cholecystitis. Nasogastric suction was systematically used to aspirate gastric content during the operation and tubes were removed before patients were sent to the recuperation ward.

A drain was placed in 115 patients whose clinical record number was even and no drain was left in the remaining 85. The drain was a polyvinyl catheter with an internal diameter of 1.6 mm and an external of 2.8 mm, with multiple perforations at its end. It was brought out through a stab wound 3 cm below the main incision. A collapsible plastic receptacle was employed as sump. Drains were removed at the third postoperative day.

The postoperative management and follow up were identical in both drained and not drained groups, except for the management of the drain itself.

The statistical method employed was the student t test.

### RESULTS

The drain's output was never more than 50 ml of sero-sanguinous fluid and in none of the 115 cases was bile drained. Postoperative fever (more than 37.5°) was noted in 27 (23%) of the 115 patients with drainage in comparison with only 4 (4%) of the 85 patients without drainage ($p < 0.001$). Fever persisted for 48 hours and disappeared spontaneously and was not accompanied by any other sign or symptom.

Wound infection occurred in 8 (7%) of the patients with drainage but in none of the patients without drains ($p < 0.001$). Wound infection was arbitrarily defined as any wound that drained or required opening, and therefore included hematomas and seromas, even with negative bacteriological cultures. The incidence of wound infection in relation to the technical difficulties encountered at the operation showed no difference between the three groups (simple, difficult and very difficult cholecystectomy). In the group with drains there were 1 basal atelectasis and 1 prolonged ileus.

The median postoperative hospital stay of the patients without drainage was 3.5 days and for those with drainage, 4.7 days ($p < 0.001$). The considerations for hospital discharge were: good general condition, good tolerance to pain with oral analgesics and resumption of oral intake and stool movements.

### DISCUSSION

Drainage of the subhepatic space after biliary surgery has been traditionally advocated, but rarely its efficacy has been evaluated in prospective trails. When the gallbladder bed can be completely obliterated and there is no active suppurative process the use of drains is possibly based on old habits, as demonstrated by a survey wherein 93% of the chairmen in different American hospitals used drains routinely[8].

The major reason for drainage of the subhepatic space after cholecystectomy is the fear of bile leakage that may lead to bile peritonitis. This is usually due to an aberrant bileduct and not slippage of the cystic duct ligature. The belief that surgical drainages serve as an early warning of bile leakage, impending bile peritonitis or intra-abdominal hemorrage is nowadays in dispute. Thus the lack of bile from a drain cannot be interpreted as indicating the absence of bile leakage or the absence of impending bile peritonitis[9–10].

Elboim[10] studied echographically a series of cholecystectomies and observed an incidence of 30% of fluid collections when drains had been placed in the subhepatic space and no collection when drains were avoided. All these collections were asymptomatic and disappeared spontaneously. Van der Linden,[11] injecting erythrocytes labelled with radioactive Tc 99 through the drain after the operation, showed a very rapid absorption. So drainage is not needed to evacuate small amounts of blood and paradoxically, be least effective when most needed: when there is much intraabdominal blood.

At the beginning of this century Yates[12] demonstrated that prophylactic drains were quickly isolated and therefore prevented from performing their function. Recent experimental studies[13] showed: a) When a drain is inserted in a peritoneal cavity that contains no fluid, it is quickly surrounded by omentum and thereby isolated; b) The lumen of the tube drain is completely occluded within 48 hours by omental growth through the end and side holes; c) The damage to serosal surfaces inhibits activation of plasminogen, which lyses fibrin clots, and contributes to the isolation of drains. Nora[14] affirmed that drains allowed to remain when intraperitoneal drainage had stopped create a problem similar to prophylactic drainage. No longer does the drain serve only as an exit for fluid, but it also becomes an entry for infection by organisms considered heretofore non pathogenic. Bengmark[15] considers this possibility one of the main reasons for not draining after elective cholecystectomy.

Myers[16] described in 1962 "the drain fever syndrome" after cholecystectomy. This consisted in a syndrome of fever and pain in the right upper quadrant which usually occurred after manipulation of a drain that had been present for more than 48 hours. The pain and the fever persist for one to three days and then spontaneously subside. Fever without any apparent cause was detected in 23% of the patients with drainage and in only 4% of those without drainage. In the drained group removal of the drain usually stopped the fever. This higher incidence of fever in the group of patients with drainage, which is statistically significant, may have to do with three factors: a) Drains can stimulate a foreign body reaction[17–18]; b) Drains provide a two-way conduit for bacteria between the skin and the peritoneal cavity[14]; c) Drains may cause discomfort to the patient and an inability to cough[6]. In this study the drains were left for three days considering the possibility of a late bile leakage, which never presented.

The higher incidence of wound infection (7%) in the group with drainage was statistically significant in comparison to the group without drainage. These results are similar to those reported in retrospective series[9-19-20]. Theoretically it can be due to: a) Contamination of the incision because of the placement of a drain; b) Devitalization of cellular tissue[14]. Cruse and Foord[23] demonstrated that the incidence of wound infection was increased more than five times when drains were brought out through the wound as compared with stab incision drainage.

The mean postoperative hospitalization for the present study was shorter than any other other series reported to date[21-22]. For the 85 patients without drainage it was 3.5 days and of 4.7 for those with drains, a difference which is statistically significant. The shorter hospitalization of the patients without drainage represents an unbiased judgement of a better hospital course. The potential advantage of shorter hospitalization is obvious.

### *References*

1. Langenbuch, C. (1882) Ein fall con Extirpation der Gallenblase wegen Chronischer Cholelithiasis. *Heilung. Berl. Klin. Wochenschr.*, **19**, 725–7
2. Kole, W. (1969) Erfaungen mit der Drainagelosen, idealen Cholecystektomie. *Langenbecks Arch. Chir.*, **324**, 307–11
3. Verbrycke, J.R. (1927) Cholecystectomy without drainage: report of eighty-six cases without mortality. *Med. J. Rec.*, **126**, 705–7
4. Fowler, R.S. (1931) Cholecystectomy without drains. *Ann. Surg.*, **93**, 745–8
5. Dreese, W.C. (1963) Cholecystectomy without drains. *J. Int. Coll. Surg.*, **40**, 433–7
6. Kambouris, A.A., Carpenter, M.S. and Allaben, R.D. (1973) Cholecystectomy without drainage. *Surg. Gynecol. Obstet.*, **137**, 613–7
7. Williams, C.B., Halpin, D.S. and Knox, J.S. (1972) Drainage following cholecystectomy. *Br. J. Surg.*, **59**, 293–6
8. Budd, D.C., Cochran, R.C. and Fouty, W.J. (1982) Cholecystectomy with and without drainage. A randomized study of 300 patients. *Am. J. Surg.*, **143**, 307–10
9. Goldberg, I.M., Goldberg, J.P., Liechty, R.D. *et al.* (1975) Cholecystectomy with and without drainage. *Am. J. Surg.*, **130**, 29–32
10. Elboim, C.M., Goldman, L., Hann, L. *et al* (1983) Significance of post-cholecystectomy subhepatic fluid collections. *Ann. Surg.*, **198**, 137–141
11. Van der Linden W., Kempi, V. and Gedda, S. (1981) A radionuclide study on the effectiveness of drainage after elective cholecystectomy. *Ann. Surg.*, **193**, 155–160
12. Yates, J. (1905) An experimental study of the local effects of peritoneal drainage. *Surg. Obstet. Gynecol.*, **1**, 473–6
13. Agrama, H.M., Blackwood, J.M., Brown, C.S. *et al.* (1976) Functional longevity of intraperitoneal drains. *Am. J. Surg.*, **132**, 416–21
14. Nora, P.F., Vanecko, R.M. and Brasfield, J.J. (1972) Prophylactic abdominal drains. *Arch. Surg.*, **105**, 173–6
15. Bengmark, S. (1988) Personal communication
16. Myers, M.B. (1962) Drain fever: a complication of drainage after cholecystectomy. *Surgery*, **52**, 314–8
17. Glenn, F. (1975) Trends in surgical treatment of calculous disease of the biliary tract. *Surg. Gynecol. Obstet.*, **140**, 877–81
18. Reynolds, J.T. (1955) Intraperitoneal drainage: when and how. *Surg. Gynecol. Obstet.*, **101**, 242–6
19. Maull, K.I., Daugherty, M.E., Russell Shearer, G. *et al.* (1978) Cholecystectomy: to drain or not to drain. A randomized study of 200 patients. *J. Surg. Res.*, **24**, 259–63
20. Todd, G.J., Reemstma, K. (1978) Cholecystectomy with drainage. *Am. J. Surg.*, **135**, 622–3
21. Trowbridge, P.E.A. (1982) Randomized study of cholecystectomy with and without drainage. *Surg. Gynecol. Obstet.*, **155**, 171–4

22. Edlund, G., Gedda, S. and Van der Linden, W. (1979) Intraperitoneal drains and nasogastric tubes in elective cholecystectomy. A controlled clinical trial. *Am. J. Surg.*, **137**, 775–9
23. Cruse, P.J. and Foord, R. (1973) A five year prospective study of 23.649 surgical wounds. *Arch. Surg.*, **107**, 206–13

(Accepted by S. Bengmark on 10 October 1989)

### INVITED COMMENTARY

The Authors of this paper have correctly identified that the issue regarding the use of routine drainage following cholecystectomy has never been satisfactorily resolved. One must therefore ask the question why. In a recent Editorial by Alexander-Williams he suggests that this issue persists because of a polarization of surgical opinion[1]. On the one side are the “ironmasters” who do and teach didactically. On the other hand are the “eggheads” who address such questions with the aid of randomized trials. I had hoped that this study would fall into the latter category and answer his plea for a properly conducted study in an attempt to end the conflict. Alas, in some important respects this is not the case. No study has yet been published that did not contain one of the following major deficiencies — small numbers, retrospective design, selected cases, preoperative randomization or uncontrolled randomization. The present study also fails on a number of these counts. Randomization was prior to surgery and there is no doubt that this may lead to an element of operator bias. Disappointingly, only elective cases were included and the overall patient numbers were small resulting in a significant imbalance between the size of study groups. These problems mean that in essence this work does not substantially aid in the solution of this dilemma as it is these very deficiencies of study design that has allowed the controversy to prosper.

However, the authors do raise a number of important points and their work does add weight to the growing data concerning drainage following cholecystectomy.

The usual reason stated for using a drain is the fear of a massive subhepatic collection of bile or blood in a review of 1546 cases the percentage of patients requiring relaparotomy for bile collection was only 0.26%[2]. Indeed it is possible to find many series particularly within the last 15 years where no such cases are reported. It is therefore likely that the fear of subhepatic bile collections has been overestimated in the past and that with operative cholangiography and careful surgical technique the number of cases will be kept to a minimum. Of course, in order to demonstrate a significant difference in the incidence of bile peritonitis following cholecystectomy, more than 40,000 patients would have to be entered into each study group if one accepts an incidence of 0.2% — surely an impractical proposition. This would tend to support the suggestion from Baraldi that clinically significant bile leakage is due to technical error and not an inevitable feature of performing large numbers of cholecystectomies[3]. If this is the case then it is unlikely that the presence of a drain would make any difference to the outcome.

Furthermore, as the Authors suggest many patients may leak small quantities of bile into the subhepatic space following cholecystectomy as suggested by several studies using radiolabelled agents.

The next question is whether the drain will work once inserted. In the published literature, the majority of the cases requiring reoperation for bile peritonitis were drained. In collected series of studies where reoperation for bile peritonitis is reported, of a total of 1277 patients, 16 underwent re-exploration for bile collections — all were drained[2,4–8]. In these studies, no undrained patients were re-explored. Clearly the drain failed to work.

Data such as this has fuelled the contention that the drains may actually be harmful and therein lies the real question. In addition to the wide range of complications uniquely associated with the use of a drain such as bleeding from the insertion site and drain migration, this series has confirmed the association between the use of drains and an increased morbidity rate. However, is there a possibility that the drains may actually cause bile leaks? Suggested causes for this are irritation due to the foreign material of the drain, prevention of tissue tamponade and the unknown effects of vacuum suction from the drain. It may be that selected "high risk and difficult" cases from whom drainage is often reserved are the very cases in whom a drain should be avoided. At this time there is no experimental or clinical evidence to support or refute this contention and this intriguing question remains unanswered.

In conclusion, while this study does attempt to consider an issue in a rational fashion, I feel the Authors have allowed themselves fall into the trap like many before them. This issue will only be resolved by a tightly controlled study that avoids all of the potential faults in study design. I look forward to that day with anticipation.

John Monson MD, FRCS, FRCSI
Assistant Director
Academic Surgical Unit
St. Mary's Hospital Medical School
London W2 1NY, U.K.

*References*

1. Alexander-Williams J. (1980) Cholecystectomy: ironmasters and eggheads. *J. Roy. Soc. Med.*, **81**, 560–561
2. Kassum, D.A., Gagic N.M., Menon, G.T. (1979) Cholecystectomy with and without drainage. *Can. J. Surg.*, **22**, 358–360
3. Baraldi, V., Macellari, G., David, P. (1980); Cholecystectomy without drainage: a dilemma? *Am. J. Surg.*, **140**, 658–659
4. Budd, D.C., Cochran, R.C., Fouty, W.J. (1982) Cholecystectomy with and without drainage. *Am. J. Surg.*, **143**, 307–309
5. Farha, G.J., Chang, F.C. Matthews, E.H. (1981) Drainage in elective cholecystectomy. *Am. J. Surg.*, **142**, 678–680.
6. Truedson, H. (1983) Cholecystectomy with intraperitoneal drain. *Acta. Chir. Scand.* **149**, 179–185.
7. Truedson, H. (1983) Cholecystectomy with and without intraperitoneal drain. *Acta. Chir. Scand.* **149**, 393–399
8. Truedson, H., Stjernberg, N. (1983) Postoperative pulmonary ventilation after cholecystectomy with and without peritoneal drain. *Acta. Chir. Scand.* **149**, 401–406

